# Multinomial model and zero-inflated gamma model to study time spent on leisure time physical activity: an example of ELSA-Brasil

**DOI:** 10.11606/S1518-8787.2017051006882

**Published:** 2017-08-03

**Authors:** Aline Araújo Nobre, Marilia Sá Carvalho, Rosane Härter Griep, Maria de Jesus Mendes da Fonseca, Enirtes Caetano Prates Melo, Itamar de Souza Santos, Dora Chor

**Affiliations:** I Programa de Computação Científica. Fundação Oswaldo Cruz. Rio de Janeiro, RJ, Brasil; II Instituto Oswaldo Cruz. Fundação Oswaldo Cruz. Rio de Janeiro, RJ, Brasil; III Escola Nacional de Saúde Pública. Fundação Oswaldo Cruz. Rio de Janeiro, RJ, Brasil; IV Faculdade de Medicina. Universidade de São Paulo. São Paulo, SP, Brasil

**Keywords:** Motor Activity, Leisure Activities, Regression Analysis, Models, Statistical

## Abstract

**OBJECTIVE:**

To compare two methodological approaches: the multinomial model and the zero-inflated gamma model, evaluating the factors associated with the practice and amount of time spent on leisure time physical activity.

**METHODS:**

Data collected from 14,823 baseline participants in the Longitudinal Study of Adult Health (ELSA-Brasil – *Estudo Longitudinal de Saúde do Adulto* ) have been analysed. Regular leisure time physical activity has been measured using the leisure time physical activity module of the International Physical Activity Questionnaire. The explanatory variables considered were gender, age, education level, and annual *per capita* family income.

**RESULTS:**

The main advantage of the zero-inflated gamma model over the multinomial model is that it estimates mean time (minutes per week) spent on leisure time physical activity. For example, on average, men spent 28 minutes/week longer on leisure time physical activity than women did. The most sedentary groups were young women with low education level and income

**CONCLUSIONS:**

The zero-inflated gamma model, which is rarely used in epidemiological studies, can give more appropriate answers in several situations. In our case, we have obtained important information on the main determinants of the duration of leisure time physical activity. This information can help guide efforts towards the most vulnerable groups since physical inactivity is associated with different diseases and even premature death.

## INTRODUCTION

Most biological parameters and medical conditions – for example, body mass index, blood pressure, and physical activity – are originally measured on a continuous scale. If the outcomes are continuous, some type of categorisation is commonly adopted decided based on other studies. These include internationally used cut-off points or data distributions, such as medians, quartiles, and plus or minus one or two standard deviations from the mean^3^. This strategy can lead to classification errors, especially for subjects that are borderline between cut-off points, thus decreasing the value of the data originally collected^3,20^. While information loss resulting from the process of categorisation or dichotomisation can lead to similar individuals being allocated to different groups, conversely, strata considered homogeneous may comprise markedly different individuals^4,14^. Furthermore, when using continuous data, it is essential to study the statistical distribution of the variable, which often diverges from the normal distribution. On the other hand, some variables are, in their origin, a mix between yes and no response, and for those who answer yes to the first question, further information is required.

In this study, leisure time physical activity was used as an example of this behaviour, as covariates associated with doing some physical versus no activity and the amount of time spent is a continuous measurement often categorised; our interest is not only in the dichotomous response (physical activity versus no physical activity), but also in the weekly duration. The usual threshold recommended – a minimum of 150 minutes/week in order to yield beneficial health impacts^7,16^ – can be difficult to achieve, and a better understanding of which covariates are associated with the increase of even a few minutes per week might be useful from a population point of view^19^.

Physical inactivity may have been responsible for some 5.3 million of the 57 million deaths recorded worldwide, in 2008^11^, and affected the occurrence of chronic noncommunicable diseases^9^. In Brazil, the frequency of individuals doing the equivalent of 150 minutes of moderate physical activity per week in 2014 was 35%^a^. Given its importance to health and wellbeing, it is appropriate to seek to understand the conditions that facilitate (or hinder) leisure time physical activity and affect its weekly duration. Appropriate statistical methods that help understand this configuration of factors can contribute to policy making and interventions to foster this habit.

Studies of the practice of leisure time physical activity generally find persons who exercise regularly and others who do not exercise. In addition, the weekly duration of such activities varies greatly. Accordingly, the distribution of total time spent on leisure time physical activity tends to be non-negative and right-skewed, in addition to displaying excess zeros (persons who do no physical activity). This type of data, known as semi-continuous or zero-inflated data, is common in research in various fields. The commonest approach to modelling this type of distribution is to categorise the variable into two or three groups and to fit logistic or multinomial regression models^8,15^. However, as the variable is primarily continuous, it is possible that important information may be lost in the process of categorisation^3^. One alternative is to construct a two-part model: one considering the likelihood of an individual engaging in leisure time physical activity regularly or not exercising at all, while the other considers the duration of such activity. This can be done using a mixed model, which combines a binomial distribution and a continuous distribution, which is in this case the gamma distribution^10^. This approach can be called the zero-inflated gamma (ZIG) model. As the value zero can only come from the binomial distribution (the gamma distribution is asymmetrical and strictly positive), the factoring of the likelihood allows the model to be fitted separately. These models are known as two-part or “hurdle” models, in which zeros and non-zeros are considered as two independent processes^13^.

This study aimed to compare two methodological approaches: the multinomial model and the zero-inflated gamma model, evaluating the factors associated with the practice and amount of time spent on leisure time physical activity.

## METHODS

### The ELSA-Brasil Study

The study population comprised the 2008–2010 baseline participants of the *Estudo Longitudinal de Saúde do Adulto* (Longitudinal Study of Adult Health, ELSA-Brasil), a multicentre cohort study involving civil servants from six teaching and research institutions in different cities in Brazil, whose main objectives were to estimate the incidence of cardiovascular diseases and diabetes and the main social, environmental, occupational, and biological determinants of the participants. Details of the study can be found in other publications^2,21^.

The 2008–2010 study baseline comprised 15,105 retired and active civil servants aged from 35 to 74 years. Of those, our analyses in this study included 14,823 participants with complete data on all the variables of interest.

Regular leisure time physical activity was measured using the leisure time physical activity module of the International Physical Activity Questionnaire (IPAQ)^5^. The questions cover the weekly frequency and duration of walking and moderate- or vigorous-intensity physical activities engaged in for 10 minutes at least during leisure time. For purposes of analysis, an approximate mean for the regularity of the leisure time physical activity was obtained by multiplying the number of days when physical activity was done by its duration in minutes.

To evaluate the two approaches we used the following covariables: gender, age (in years), education level (basic education, high school, college, graduate), and annual *per capita* family income, calculated as the midpoint of the net income category reported divided by the number of persons dependent on that income. In order to facilitate interpretation of the coefficients of the models, age was recorded in 10-year units and annual *per capita* family income in US$1,000.

### Statistical Analysis

Two approaches were used: the multinomial model, which is applied when the outcome is categorical with more than two levels, and the zero-inflated gamma model, which considers the variable in its original form. For the first approach, the measure of association estimated for each independent variable was the odds ratio and, for the second one, in addition to odds ratios, mean differences in time spent on leisure time physical activity were also estimated.

#### Multinomial model

For the multinomial model, the categorisation used was: no leisure time physical activity, less than 150 minutes/week, and 150 minutes/week or more (cut-off point recommended recently for promoting and maintaining adult health)^7,16^. Physical inactivity was taken as the reference category.

#### Zero-inflated gamma model

Using this model, both facts can be contemplated: that various factors influence the decision to do, or not to do, leisure time physical activity and that the time devoted to leisure time physical activity may be associated with different factors. Let *Y*_i_ be total time, in minutes per week, spent on leisure time physical activity by individual *i*, and *X*_i_, a binary variable with binomial distribution, with *X*_i_ *= 1* indicating that the individual *i* does leisure time physical activity and *X*_i_ *= 0*, no leisure time physical activity. Thus, *P (X*_i_ *= 1) = p*_i,_ where *p*_i_ is the probability of individual *i* doing leisure time physical activity. If a given individual practices leisure time physical activity, its duration will be modelled using a gamma distribution with mean *µ*_i_ and variance *µ*_i_^2^ ν^2^. The likelihood function of the model is given by:

Lp,μ,ν=LpLμ,ν=ΠYi=01-piΠYi>0piΠYi>0fyiμi, v

This factoring of the likelihood allows the models to be fitted separately. In order to model the probability of doing leisure time physical activity, *p*_i_, we used a logistic regression such that *logit(p*_i_ *) = x*_i_^’^α, where *x*_i_ represents the covariables and α, the respective parameters. In order to model the duration of leisure time physical activity, we used a gamma regression. The inverse function is the canonical link function of a gamma distribution in a generalized linear model^10^. However, the coefficients are difficult to interpret. As an alternative, this study used the identity link function, such that *µ*_i_ *= w*_i_ *’*β, making the coefficients easy to interpret. The combination of these two parts of the model, as set out in equation 1, yields a zero-inflated gamma (ZIG) distribution. Note that the covariables used in each part of the model are not necessarily the same. The analyses were performed using software R version 3.2.2^17^.

## Ethical Considerations

The study was approved by the National Research Ethics Commission (CONEP – 976/2006) and by the Research Ethics Committee of each institution. All participants signed an informed consent form.

## RESULTS

Mean age of participants was 52 years (with standard deviation of 9.1 years), 54.4% were women, 36.8% had graduate education, and mean annual *per capita* income was US$6,505. Of the participants, 6,369 (43%) did no leisure time physical activity and, among those who did, mean time spent on leisure time physical activity was 237 minutes/week. As shown in the Figure, the distribution of the duration of leisure time physical activity is quite skewed, as it displays excess zeros, and only a few participants reported doing more than 1,000 minutes of leisure time physical activity per week. Physical inactivity was reported more frequently by women (48.0%), participants with lower education level (55.4%), and the group with lower mean age and *per capita* income. Men who did engage in physical activity on average spend more time than women. We did not observe a clear gradient between education level and total time spent on physical activity (Table 1).

**Figure:**
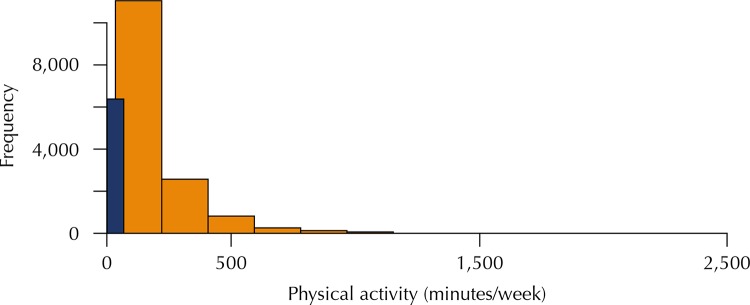
Histogram of the distribution of total time spent on physical activity by participants in ELSA-Brasil, 2008–2010.

**Table 1:** Total time spent on physical activity by population characteristics. ELSA-Brasil, 2008–2010.

Variable	n	Physical activity (min/week)			

		no	< 150	≥ 150	Total time spent
Mean age (years)	-	51.5	52.3	52.8	-
Mean annual *per capita* income (US$)	-	5,318.30	6,903.11	7,703.00	-
Gender					
Women	8,061	48.0	20.5	31.5	224.7
Men	6,762	36.9	23.0	40.1	249.0
Education level					
Basic	1,884	55.4	20.2	24.4	220.3
High school	5,125	51.9	19.5	28.6	233.9
College	2,351	30.5	24.4	45.2	242.8
Graduate	5,463	42.5	21.3	36.2	240.5

Table 2 shows the odds ratios estimated from the multinomial model and the logistic part of the zero-inflated gamma model, as well as the coefficients estimated by gamma regression and their 95% confidence intervals. The variables age, gender, and *per capita* income were significant in all the models. In the multinomial model, at each 10-year increase in the age of participants, the adjusted odds of doing leisure time physical activity for more than 150 minutes/week and up to 150 minutes/week increased 1.14 and 1.07 times, respectively. This type of estimate was obtained for each of the other variables of interest.

**Table 2:** Results of the multinomial and zero-inflated gamma models for total time spent on physical activity. ELSA-Brasil, 2008–2010.

Variable	Multinomial^a^		Zero-inflated gamma	
	
	< 150 min/week	≥ 150 min/week	Logistic^a^	Gamma^b^
			
	OR (95%CI)	OR (95%CI)	OR (95%CI)	Coeff. (95%CI)
Age in 10-year intervals	1.07 (1.06–1.09)	1.14 (1.13–1.15)	1.12 (1.07–1.16)	6.20 (1.47–10.97)
Gender				
Men	1.54 (1.41–1.68)	1.81 (1.67–1.95)	1.70 (1.58–1.82)	28.78 (20.24–37.35)
Education level				
High school	1.11 (0.99–1.24)	1.41 (1.26–1.58)	1.28 (1.14–1.43)	19.93 (4.68–34.62)
College	1.40 (1.22–1.60)	2.00 (1.75–2.28)	1.72 (1.51–1.97)	25.81 (8.30–43.09)
Graduate	1.87 (1.65–2.12)	2.69 (2.38–3.05)	2.32 (2.04–2.63)	14.45 (–1.57–29.93)
Annual *per capita* income in US$1000	1.04 (1.03–1.05)	1.06 (1.05–1.06)	1.05 (1.04–1.06)	7.58 (2.91–20.50)

^a^ Reference category – does no physical activity.

^b^ Significance of parameters evaluated compared with zero value.

In addition to the information that men are 70% more likely to engage in leisure time physical activity than women, the ZIG model also informed that, among the participants who did leisure time physical activity, after adjusting for age, education level, and income, men exercised for a mean 28 minutes more per week than women. The logistic part and multinomial model returned a gradient in which the higher the education level, the greater the likelihood of doing leisure time physical activity. Even though that gradient was not observed in the gamma part of the ZIG model, the coefficients for education at the levels of high school and college are significant and suggest more time spent on leisure time physical activity as compared with participants who had only basic education. At each US$1,000 increase in the participants’ annual *per capita* income, the adjusted likelihood of doing leisure time physical activity was 1.05 times greater and they approximately spent a mean of seven minutes more per week on leisure time physical activity. Similar results were found in the multinomial model; the adjusted odds of doing leisure time physical activity for more than 150 minutes/week and up to 150 minutes/week increased 1.06 and 1.04 times, respectively.

## DISCUSSION

In this application, the analysis of the time spent on leisure time physical activity demonstrated the usefulness of the gamma distribution for fitting outcomes that are primarily continuous and skewed. The presence of zeros, which in this case indicate physical inactivity, led us to construct a model that combined the gamma and binomial distributions. As the weekly duration of leisure time physical activity has an important protective effect with regard to numerous health outcomes, it is extremely important to be able to discover not only whether or not leisure time physical activity is done, but also some of the main determinants of the duration of such leisure time physical activity for public health purposes^9^. The multinomial model estimates only the probability of leisure time physical activity being done, by category of variable, as compared with “not being done”, for example^10^. In that type of strategy, the mean time spent on the activity (in minutes per week) cannot be estimated by converting time spent on leisure time physical activity into a categorical variable. That is the main advantage of using the zero-inflated gamma model. On the other hand, investigators have postulated that even lower levels of physical activity may be associated with health benefits^24^. This dose-response relationship is more easily captured by the multinomial model.

The two-part regression model, in which zeros may occur in only one of the parts, is a simple and statistically valid methodology to analyse skewed data with zeros. Ribeiro et al.^18^, in their study of leisure time physical activity, has divided the research question into two components: participation in and time spent on leisure time physical activity. However, the weekly duration of leisure time physical activity has been modelled by means of a normal distribution, which is a symmetric probability distribution and generally does not correspond to the distribution of leisure time physical activity. In our approach using gamma distribution, the models were separated by factoring the likelihood.

With the ZIG model, the variables that are associated with the outcome in each part of the model can be different. For example, in our study, only the participants with high school and college education displayed significant increases in time spent on leisure time physical activity compared with participants who had only basic schooling. Meanwhile, the decision to practice or not leisure time physical activity was associated with all education levels. In addition, by using the identity link function in the gamma regression, we could interpret the model parameters directly^12^. For example, men exercised, on average, 28 minutes/week more than women.

The use of questionnaires to assess physical activity, including the IPAQ, is an imprecise method. The time spent on physical activity is usually overestimated and has been recently replaced by objective measures^6,23^. As the focus of the article was to compare models based on the same data set, limitations related to data acquisition do not affect the conclusions. Usual model comparison criteria such as Akaike information criterion (AIC)^1^ and Bayesian information criterion (BIC)^22^ were not used because they do not exactly model the same response variable.

We consider that the ZIG model, which is rarely used in epidemiological studies, can offer more appropriate answers in a variety of situations. The models fitted in this study gave substantial information on the problem of physical inactivity, making it possible to quantify the association with important variables, such as gender, age, education level, and income. Given that physical inactivity is associated with different diseases and even premature death, this information can help direct efforts and policies towards the most vulnerable groups. In that regard, the emphasis should be on the most sedentary – a group comprising young women with low education level and income.

## Footnotes

a Ministério da Saúde (BR), Secretaria de Vigilância em Saúde, Departamento de Vigilância de Doenças e Agravos não Transmissíveis e Promoção da Saúde. VIGITEL Brasil 2014: vigilância de fatores de risco e proteção para doenças crônicas por inquérito telefônico. Brasília (DF); 2O15 [cited 2017 Mar 10]. Available from: http://bvsms.saude.gov.br/bvs/publicacoes/vigitel_brasil_2014.pdf
